# FGF21 improves glucose homeostasis in an obese diabetes-prone mouse model independent of body fat changes

**DOI:** 10.1007/s00125-017-4389-x

**Published:** 2017-08-02

**Authors:** Thomas Laeger, Christian Baumeier, Ilka Wilhelmi, Josefine Würfel, Anne Kamitz, Annette Schürmann

**Affiliations:** 1 Department of Experimental Diabetology (DIAB), German Institute of Human Nutrition Potsdam-Rehbruecke (DIfE), Arthur-Scheunert-Allee 114-116, 14558 Nuthetal, Germany; 2 German Center for Diabetes Research (DZD), München-Neuherberg, Germany

**Keywords:** Energy expenditure, FGF21, Glucose homeostasis, Insulin resistance, NZO, Obesity, Thermogenesis, UCP1

## Abstract

**Aims/hypothesis:**

Fibroblast growth factor 21 (FGF21) is considered to be a promising therapeutic candidate for the treatment of type 2 diabetes. However, as FGF21 levels are elevated in obese and diabetic conditions we aimed to test if exogenous FGF21 is sufficient to prevent diabetes and beta cell loss in New Zealand obese (NZO) mice, a model for polygenetic obesity and type 2 diabetes.

**Methods:**

Male NZO mice were treated with a specific dietary regimen that leads to the onset of diabetes within 1 week. Mice were treated subcutaneously with PBS or FGF21 to assess changes in glucose homeostasis, energy expenditure, food intake and other metabolic endpoints.

**Results:**

FGF21 treatment prevented islet destruction and the onset of hyperglycaemia, and improved glucose clearance. FGF21 increased energy expenditure by inducing browning in subcutaneous white adipose tissue. However, as a result of a compensatory increased food intake, body fat did not decrease in response to FGF21 treatment, but exhibited elevated *Glut4* expression.

**Conclusions/interpretation:**

FGF21 prevents the onset of diet-induced diabetes, without changing body fat mass. Beneficial effects are mediated via white adipose tissue browning and elevated thermogenesis. Furthermore, these data indicate that obesity does not induce FGF21 resistance in NZO mice.

**Electronic supplementary material:**

The online version of this article (doi:10.1007/s00125-017-4389-x) contains peer-reviewed but unedited supplementary material, which is available to authorised users.

## Introduction

Type 2 diabetes mellitus is a complex disease characterised by insufficient secretion of insulin from pancreatic beta cells (beta cell failure) in the setting of insulin resistance. Before the onset of type 2 diabetes beta cells start to proliferate and increase the biosynthesis and secretion of insulin to compensate for peripheral insulin resistance and glucose intolerance [1]. Eventually, due to a decline in the secretory rate and a decrease in beta cell mass, impaired insulin secretion fails to compensate, resulting in hyperglycaemia, beta cell destruction and type 2 diabetes. Causes of beta cell loss are chronic hyperglycaemia (glucotoxicity) and chronically elevated NEFA and lipid intermediates (lipotoxicity) [2]. Therefore, current research efforts are focused on new strategies to improve insulin sensitivity and protect beta cells against glucolipotoxicity.

Fibroblast growth factor (FGF) 21 is an endocrine hormone that has, besides its primary function of maintaining the energy homeostasis, beneficial effects on glucose homeostasis, including weight loss. Circulating FGF21 is primarily derived from the liver, but is also expressed in the gut, brain, adipose tissue, muscle and pancreas [3–5]. FGF21, which is widely considered to be induced by fasting, is the first known endocrine signal that is activated by protein restriction rather than energy deprivation [3, 4, 6]. Although FGF21 is robustly elevated in low-protein environments, increased FGF21 is also seen in various other contexts such as fasting, overfeeding, ketogenic diets and high-carbohydrate diets [7]. FGF21 signals through a cell-surface receptor complex composed of both the traditional FGF receptor (FGFR) 1c and the FGF co-receptor β-klotho [8]. Many studies have revealed the critical role of β-klotho as a cofactor essential for FGF21-mediated signalling [8–11].

FGF21 targets a variety of different organs. As recently reviewed in [12, 13], exogenously administered FGF21 acts directly on adipose tissue to reduce blood glucose levels and increase hepatic, and possibly peripheral, insulin sensitivity, decrease body weight and decrease serum lipids [14]. In adipocytes, FGF21 induces GLUT1 expression and glucose uptake [15]. FGF21 treatment activates browning of white adipose tissue (WAT), and increases thermogenesis in brown adipose tissue (BAT) and WAT [16]. Administration of FGF21 directly into the brain has been shown to induce activation of BAT, and to increase energy expenditure (EE) and insulin sensitivity [17, 18]. In the pancreas, FGF21 has been shown to improve beta cell function and survival [19], suppress islet glucagon secretion [20] and prevent pancreatic inflammation [21]. FGF21 stimulates insulin secretion in ex vivo islets isolated from rodent models of diabetes [19], but does not affect insulin secretion from islets isolated from healthy mice [22]. In the liver, FGF21 is reported to increase NEFA turnover and to reduce lipogenesis and glucose output, as recently summarised in [18].

Its beneficial roles in regulating insulin sensitivity and glucose homeostasis make FGF21 a promising therapeutic candidate for the treatment of diabetes [23]. However, obesity is thought to be an FGF21-resistant state because of the elevated endogenous FGF21 levels [24, 25]. Serum FGF21 concentrations were elevated in obese and diabetic rodent models in comparison with lean controls [20, 25, 26]. In addition, in relation to normal lean individuals, FGF21 was up to 2.0-fold higher in people with obesity and type 2 diabetes [27, 28]. Obese monkeys display high levels of FGF21 and reduced levels of β-klotho in adipose tissue, whereas monkeys that maintained normal levels of β-klotho are protected against obesity [29]. Interestingly, downregulation of β-klotho expression is not the major mechanism contributing to impaired FGF21 signalling in WAT [30]. In contrast to a few studies suggesting FGF21 resistance, numerous studies [20, 26, 31, 32] conducted in obese animals, which may have impaired FGF21 signalling in response to physiological levels of FGF21, show a protective metabolic function in regard to pharmacological levels of exogenous FGF21. Hale et al [31] successfully challenged this hypothesis and concluded that an overt FGF21 resistance was not evident in mouse models of obesity and insulin resistance. Treatment of obese, hyperglycaemic, insulin-resistant and leptin-deficient B6-*ob/ob* mice with FGF21 could normalise hyperglycaemia despite markedly elevated endogenous FGF21 levels [31]. Therefore, it appears that the elevated plasma FGF21 levels under pathological conditions represent an FGF21 resistance but they are a compensatory response.

Because of this obvious contradiction, we aimed to confirm in a model for polygenic obesity and diabetes, the New Zealand obese (NZO) mouse, the beneficial effects of FGF21 that have been observed in other models. The NZO mouse displays a characteristic trait of beta cell loss [33] in contrast to other obese mouse models, e.g. B6-*ob/ob* mice are rescued by beta cell hyperplasia caused by a chronic glucose challenge (i.e. they are diabetes resistant). Here we investigate the effects of exogenous administration of FGF21 to obese and insulin-resistant NZO mice, and evaluate the potential of the diabetes-susceptible NZO mouse as an animal model to study endogenous FGF21 actions in regard to the prevention of diabetes in the future.

## Methods

### Animals and diets

All procedures involving animals were approved by the animal welfare committees of the German Institute of Human Nutrition (DIfE) and the local authorities (Landesamt für Umwelt, Gesundheit und Verbraucherschutz, Brandenburg, Germany). The NZO/HIBomDife mice (German Institute of Human Nutrition, Nuthetal, Germany) were single-housed in 12:12 h light:dark cycle (lights on at 06:00 hours) at a temperature of 21 ± 1°C with ad libitum access to food and water unless otherwise noted. To investigate if the plasma FGF21 levels correlate with the blood glucose levels in NZO mice, male NZO mice received (after weaning at 3 weeks of age) a high-fat diet containing 45% (of total energy) fat, 35% carbohydrate and 20% protein (D12451, Research Diets, New Brunswick, NJ, USA). To test the ability of FGF21 to prevent diabetes we used another group of mice and treated them with a specific dietary regimen that leads to the onset of diabetes within 1 week [33–36]. Male NZO mice received (after weaning at 3 weeks of age) a standard chow diet for 2 weeks. At 5 weeks of age, mice were placed on a carbohydrate-free high-fat (−CH) diet (32% [wt/wt] protein, 0% [wt/wt] carbohydrate and 31% [wt/wt] fat; 16.8 kJ/g; #105789, Altromin, Lage, Germany) for 13 weeks, at which point a random subgroup of animals was transferred to a carbohydrate-containing high-fat (+CH) diet (20% [wt/wt] protein, 40% [wt/wt] carbohydrate and 28% [wt/wt] fat; 21.9 kJ/g). A detailed dietary composition is provided in the electronic supplementary material (ESM) Table 1. At the end of this study, mice were killed during the mid-light cycle in a 4 h fasted state using acute exposure to isoflurane followed by rapid exsanguination via vena cava blood collection. Blood was centrifuged at 10,000 *g* at 4°C for 10 min. Tissues were collected and snap-frozen in liquid nitrogen for further analysis.

### Experimental design

At 5 weeks of age, male NZO mice were placed on a −CH diet for 13 weeks, at which point a random subgroup of animals was transferred to a +CH diet for 2 weeks. Animals were treated twice daily with s.c. PBS (pH 7.4) or rhFGF21 in PBS (1 μg/g bodyweight; no. CYT-474; ProSpec, East Brunswick, NJ, USA) starting 3 days before and ending 7 days after the diet switch. An OGTT was performed 8 days after the diet switch. After an overnight 16 h fasting period, mice received 2 mg glucose per g body weight by oral gavage. At the indicated points of time, blood glucose and plasma insulin were measured. Body weight and food intake were measured daily. Body composition was analysed via quantitative magnetic resonance (EchoMRI 2012 Body Composition 115 Analyzer, Houston, TX, USA) 6 days before the diet switch, at the beginning of experimental diets (day 0), on the final day of PBS or rhFGF21 treatment (day 7) and on the day of euthanisation (day 14). Random blood glucose and equivalent serum insulin levels were measured from tail blood every 2–3 days throughout the experiment. Fourteen days after the diet switch, mice fasted for 4 h were treated s.c. with PBS or rhFGF21 (1 μg/g bodyweight) 30 min before being killed. For analysis of EE and respiratory exchange ratio (RER), the transition to diet switch occurred within metabolic chambers (PhenoMaster/LabMaster; TSE Systems, Bad Homburg, Germany). Mice were treated as described before. Body weight and food intake were measured daily. Two days after the diet switch, mice fasted for 4 h were killed and tissues collected.

### Determination of FGF21 and insulin

Concentrations of mouse and recombinant human FGF21 in plasma were determined by specific ELISAs according to the procedure recommended by the manufacturer (no. RD291108200R, mouse and rat FGF-21 ELISA; no. RD191108200R, human FGF21 ELISA; BioVendor, Brno, Czech Republic) and as described previously [3, 4]. Plasma insulin concentrations were determined with an ELISA (80-INSMSU-E01; ALPCO Diagnostics, Salem, NH, USA) [37].

### Western immunoblot analysis

Western blot analysis was performed as described previously [35] using 20 μg sample solutions, 12% polyacrylamide gels, 0.45 μm pore size polyvinylidene difluoride (PVDF) membranes (Immobilon-P; Merck Millipore, Billerica, MA, USA), appropriate primary antibodies against extracellular signal-regulated kinase 1/2 (ERK1/2; 1:1000; no. 4695; Cell Signaling, Danvers, MA, USA), phospho-ERK1/2 (1:1000; no. 4377; Cell Signaling) and GAPDH (1:25,000; no. AM4300; ThermoFisher, Waltham, MA, USA), and secondary antibodies (horseradish peroxidase-conjugated anti-mouse and anti-rabbit IgG; 1:20,000). Bands were quantified using FusionCapt Advance Solo4 v16.07 software (Vilber Lourmat, Eberhardzell, Germany). The level of phosphorylation of ERK was calculated relative to the total amount of this enzyme.

### Immunohistochemistry of pancreatic islets

Pancreatic tissue excised immediately after exsanguination was fixed in 4% (w/vol.) formaldehyde and embedded in paraffin according to standard procedures. For co-staining of insulin and glucagon, mouse monoclonal anti-insulin (1:50,000; cloneK36AC10; Sigma-Aldrich, Munich, Germany) and polyclonal goat anti-glucagon (1:50; sc-7779; Santa Cruz, Dallas, TX, USA) antibodies were used. Alexa Fluor 594-labelled donkey anti-mouse (1:200; no. A-21203; ThermoFisher) and Alexa Fluor 488-labelled donkey anti-goat (1:200; no. A11055; ThermoFisher) were used as secondary antibodies. Nuclei were stained with DAPI.

### Detection of liver triacylglycerol and glycogen concentrations

Hepatic triacylglycerol content was measured using the commercial TR-210 kit (Randox, Crumlin, UK) according to the manufacturer’s protocol. Quantification of hepatic glycogen content was performed as described before [38].

### Real-time PCR

Total RNA was extracted from liver, gonadal WAT (gWAT), subcutaneous WAT (sWAT) and BAT using RNeasy Mini Kit (Qiagen, Venlo, Netherlands) following the manufacturer’s protocol. RNA purity and quantity was determined by spectrophotometry using a NanoDrop ND-1000 (VWR, Radnor, PA, USA). cDNA synthesis was performed with M-MLV reverse transcriptase (Promega, Fitchburg, WI, USA). Amplification was performed by using the LightCycler 480 II/384 (Roche, Basel, Switzerland). Expression levels were detected by applying TaqMan Gene Expression Assays (Applied Biosystems, Waltham, MA, USA). Primer assays were designed with at least one primer spanning an exon-exon boundary. Target gene expression was normalised with *Eef2* as the endogenous control.

### Statistical analysis

Data were analysed using the Prism 6 software (GraphPad Software, San Diego, CA, USA) using one-way ANOVA, two-way ANOVA or unpaired two-tailed *t* test. Analysis of EE with body weight as the covariate was assessed via ANCOVA using the MMPC.org ANCOVA data analysis tool. All data are expressed as mean ± SEM, with a probability value of 0.05 considered statistically significant. The experimenters were not blind to group assignment and no data were omitted.

## Results

### FGF21 prevents hyperglycaemia and improves glucose clearance in NZO mice

**Fig. 1:**
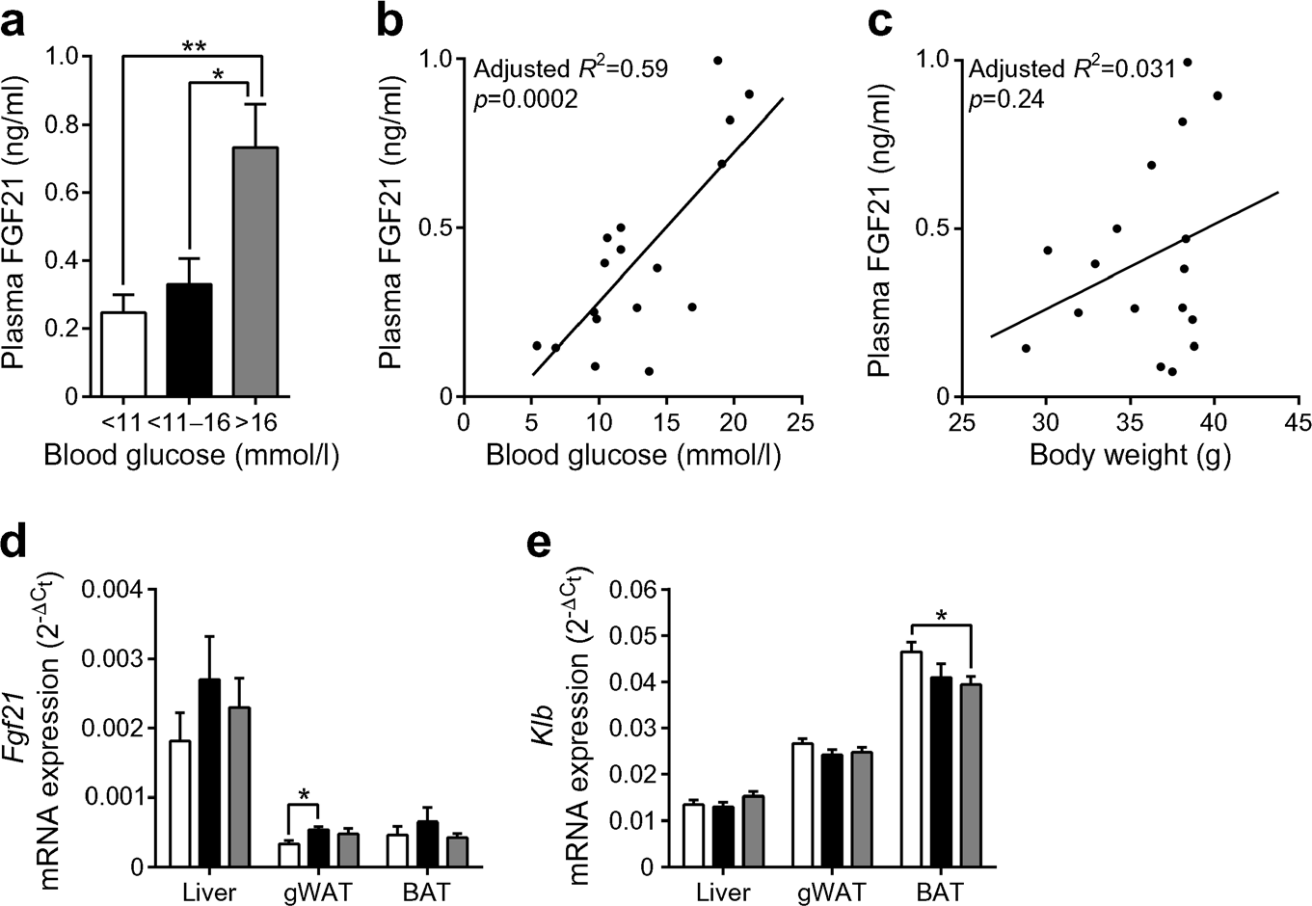
Plasma FGF21 levels correlate positively with blood glucose levels in NZO mice. Mice were placed on a high-fat diet for 3 weeks at the age of 3 weeks. (**a**) Plasma FGF21 concentrations in NZO mice that differ in blood glucose concentrations. Correlations between plasma FGF21 level and (**b**) blood glucose or (**c**) body weight. Gene expression of (**d**) *Fgf21* and (**e**) *Klb* in liver, gWAT and BAT of NZO mice. White bars, blood glucose <11 mmol/l; black bars, blood glucose 11–16 mmol/l; grey bars, blood glucose >16 mmol/l. Data are presented as mean ± SEM (*n* = 6–9/group). Differences between groups were calculated by one-way ANOVA (**a**, **d**, **e**). *p < 0.05, **p < 0.01

Obesity and diabetes are known for increasing circulating FGF21 levels. As expected, plasma FGF21 levels correlated positively with blood glucose levels in NZO mice (Fig. 1a, b) but a correlation between body weight and FGF21 levels could not be detected (Fig. 1c). The increased plasma FGF21 levels might be caused by a numerical increase of *Fgf21* mRNA expression in the liver and a significant increase in the gWAT (*p* < 0.05; Fig. 1d). The FGF21 co-receptor β-klotho (*Klb*) mRNA expression was significantly decreased in BAT (*p* < 0.05), but not affected in liver and gWAT (Fig. 1e).

**Fig. 2:**
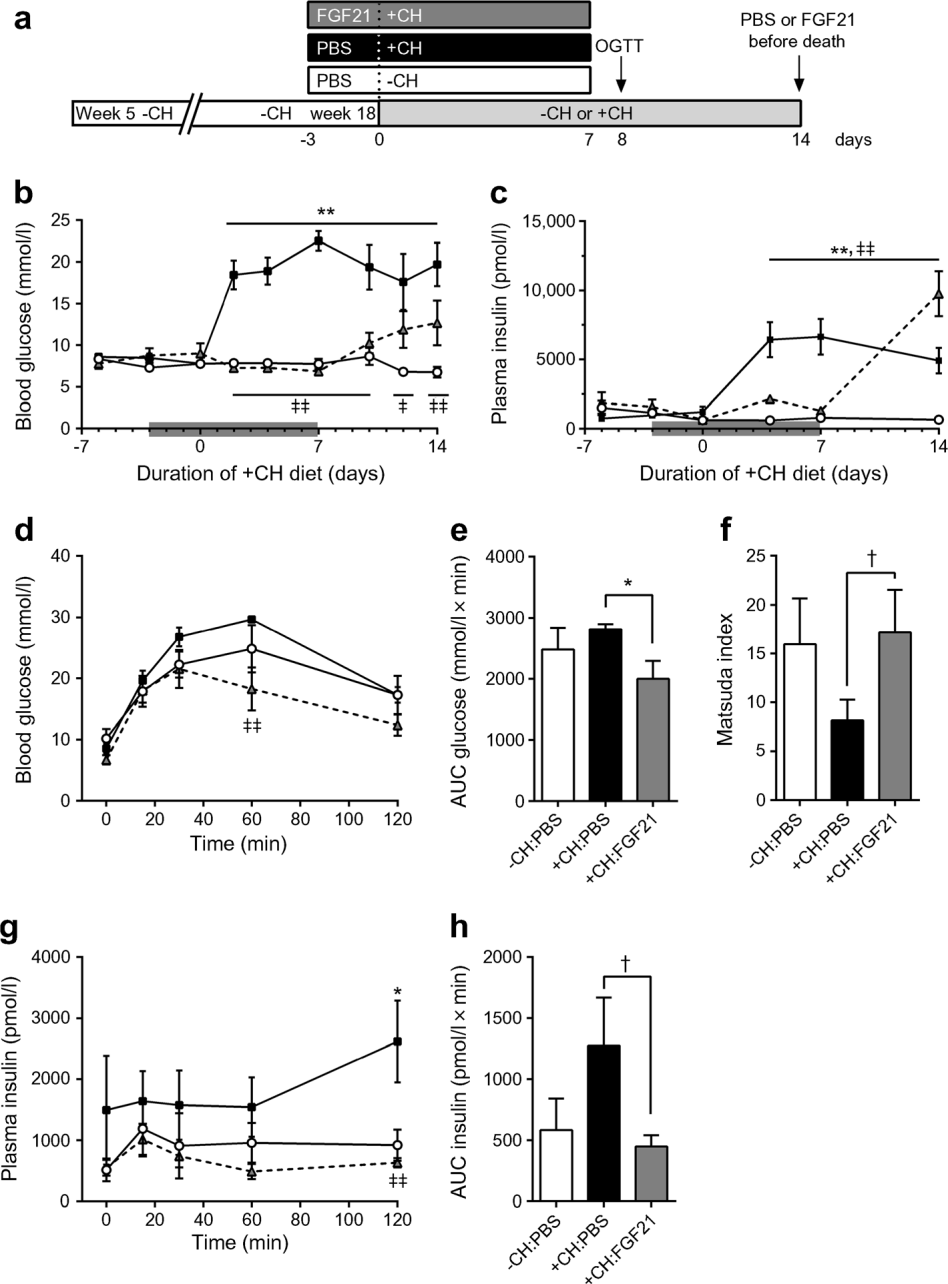
FGF21 prevents hyperglycaemia and improves glucose clearance in NZO mice. (**a**) Study design. At 5 weeks of age, NZO mice were placed on a −CH diet for 13 weeks, at which point a random subgroup of animals was transferred to a +CH diet for a number of days as indicated. (**b**) Random blood glucose and (**c**) plasma insulin in NZO mice consuming −CH or +CH diets and treated s.c. with PBS or rhFGF21 (1 μg/g body weight) starting 3 days before the diet switch and ending 7 days after the diet switch (indicated by the grey bar). Eight days after the diet switch, an OGTT was performed in mice fasted for 16 h (2 mg/g body weight glucose by oral gavage). (**d**) Blood glucose and (**e**) AUC of glucose during OGTT. (**f**) Insulin sensitivity calculated using the Matsuda Index. (**g**) Plasma insulin and (**h**) AUC of insulin during OGTT. White circles, −CH:PBS; black squares, +CH:PBS; grey triangles, +CH:FGF21. Data are presented as mean ± SEM (*n* = 6–7/group). Differences compared with +CH:PBS group were calculated by two-way ANOVA (**b**, **c**, **d**, **g**) or one-way ANOVA (**e**, **f**, **h**). ^†^0.1 > *p* > 0.05, *^,‡^ *p* < 0.05, **^,‡‡^ *p* < 0.01 (*, +CH:PBS vs −CH:PBS; ^‡^, +CH:PBS vs +CH:FGF21)

In order to test if FGF21 is sufficient to inhibit hyperglycaemia in a model of polygenetic obesity and type 2 diabetes we used NZO mice on a specific dietary regimen that leads to the onset of diabetes within 1 week [33–36]. At 5 weeks of age, NZO mice were placed on a −CH diet for 13 weeks, at which point a random subgroup of animals was transferred to a +CH diet for 2 weeks (Fig. 2a). Animals were treated s.c. with PBS or FGF21 starting 3 days before and ending 7 days after the diet switch. While the endogenous FGF21 plasma levels did not differ between the three groups before starting the applications, human FGF21 could be successfully determined by ELISA solely in the FGF21-treated group (ESM Fig. 1a, b). Consistent with previous studies [35, 36, 39], without carbohydrates NZO mice become obese and insulin-resistant, but are protected from developing diabetes. The change to the +CH diet induced a rapid hyperglycaemia (Fig. 2b). In contrast, FGF21-treated animals exhibited normal blood glucose levels similar to PBS-treated animals kept on the −CH diet (Fig. 2b). After finishing the FGF21 treatment at day 7 on the +CH diet, blood glucose levels began to rise until the end of the study. FGF21-treated +CH-fed animals displayed normal plasma insulin levels (Fig. 2c). While the plasma insulin levels rose rapidly after the change to the +CH diet in PBS-treated mice, insulin levels began to increase robustly after termination of the FGF21 treatment in +CH-fed mice (Fig. 2c). After finishing the treatment (FGF21 and PBS) an OGTT was performed. FGF21-treated mice showed improved glucose clearance (Fig. 2d, e) with low insulin levels (Fig. 2g, h). Insulin sensitivity, as determined using the Matsuda Index (Matsuda and DeFronzo [40]), was improved by trend (*p* = 0.08) in +CH-fed mice treated with FGF21 (Fig. 2f). As expected, hyperglycaemic mice showed a significant increase in hepatic *Fgf21* mRNA expression compared with normoglycaemic mice on the −CH diet (*p* < 0.05; ESM Fig. 1c) and *Klb* mRNA expression was significantly decreased in BAT, but not affected in liver, gWAT or sWAT (*p* < 0.05; ESM Fig. 1d, e).

**Fig. 3:**
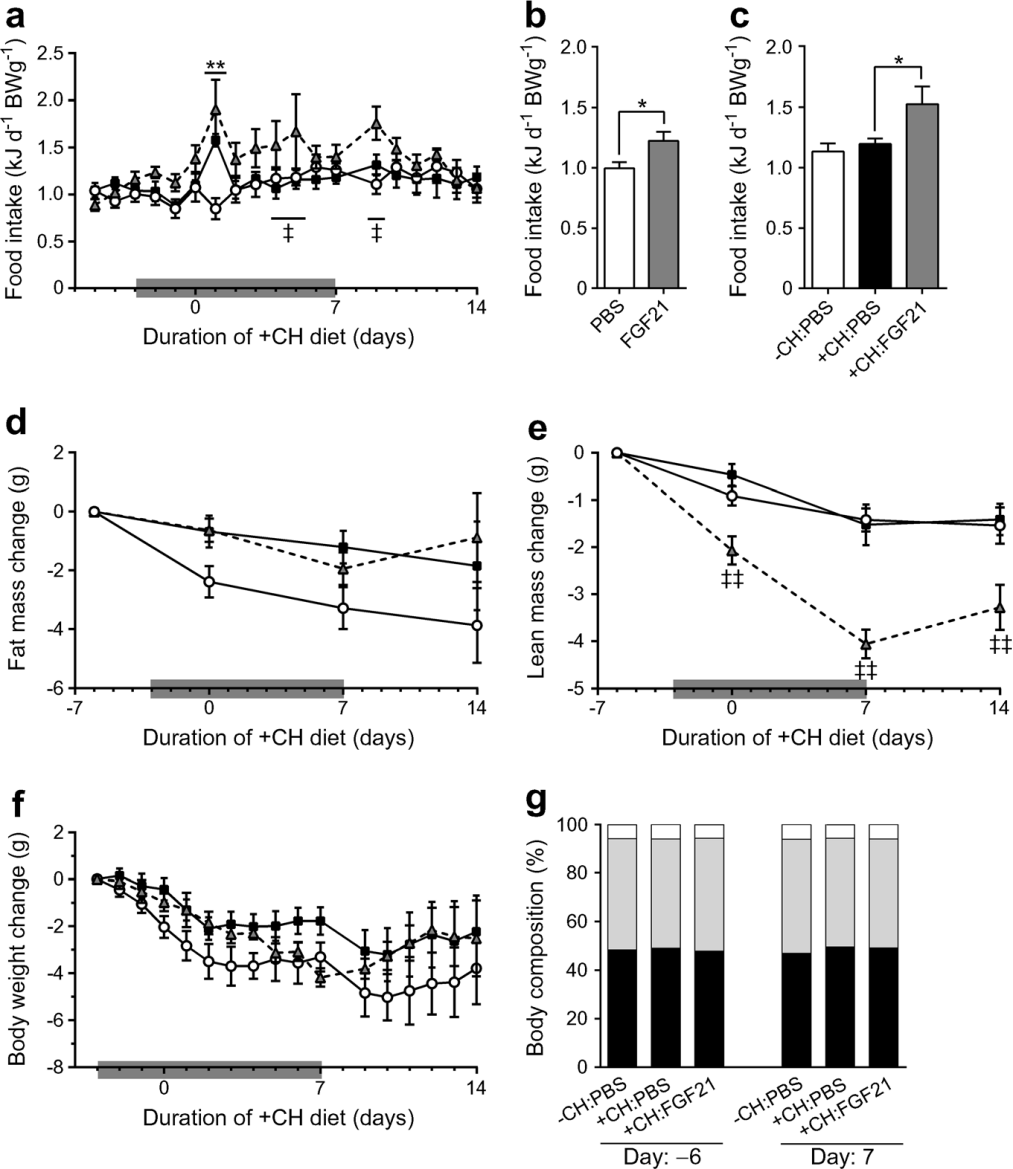
FGF21 transiently increases food intake and decreases lean mass in NZO mice. Mice were treated as described in Fig. 2. (**a**) Daily food intake and (**b**, **c**) averaged daily food intake during (**b**) the first 3 days of FGF21 treatment (day −3 until 0) and (**c**) days 1–7. Changes in (**d**) body fat mass, (**e**) body lean mass and (**f**) body weight were monitored throughout the study. (**g**) Body composition of mice before (day −6) and after (day 7) treatment with PBS or FGF21. White circles, −CH:PBS; black squares, +CH:PBS; grey triangles, +CH:FGF21. Data are presented as mean ± SEM (*n* = 6–7/group). Differences compared with +CH:PBS group were calculated by two-way ANOVA (**a**, **d**, **e**, **f**) or one-way ANOVA (**c**, **g**). Differences between PBS and FGF21 groups were analysed using a two-tailed *t* test (**b**). *^,‡^ *p* < 0.05, **^,‡‡^ *p* < 0.01 (*, +CH:PBS vs −CH:PBS; ^‡^, +CH:PBS vs +CH:FGF21)

Strikingly, FGF21 treatment induced a moderate hyperphagic response throughout the treatment independent of diet (Fig. 3a–c). At day 1 after the diet switch both PBS- and FGF21-treated NZO mice showed an increased caloric intake presumably caused by the novelty of the food (Fig. 3a). After finishing the treatment, FGF21 did not change the fat mass but decreased the lean mass by approximately 4 g (Fig. 3d–e), which led to a drop in body weight by the same extent (Fig. 3f). However, total body weight (not shown) and body weight change (Fig. 3f) did not differ between the three groups at any day throughout the experiment. Moreover, FGF21 did not affect body composition over the course of the treatment (Fig. 3g).

At the end of the study, animals fasted for 4 h were injected with PBS or FGF21, according to their former treatments, 30 min before the mice were killed to analyse ERK1/2 phosphorylation as a downstream signal for FGF21 action. As shown in ESM Fig. 1e, FGF21 treatment induced a robust increase (*p* < 0.01) in the phosphorylation of ERK1/2 in the liver, gWAT and sWAT, and a numerical increase in BAT (*p* = 0.11).

**Fig. 4:**
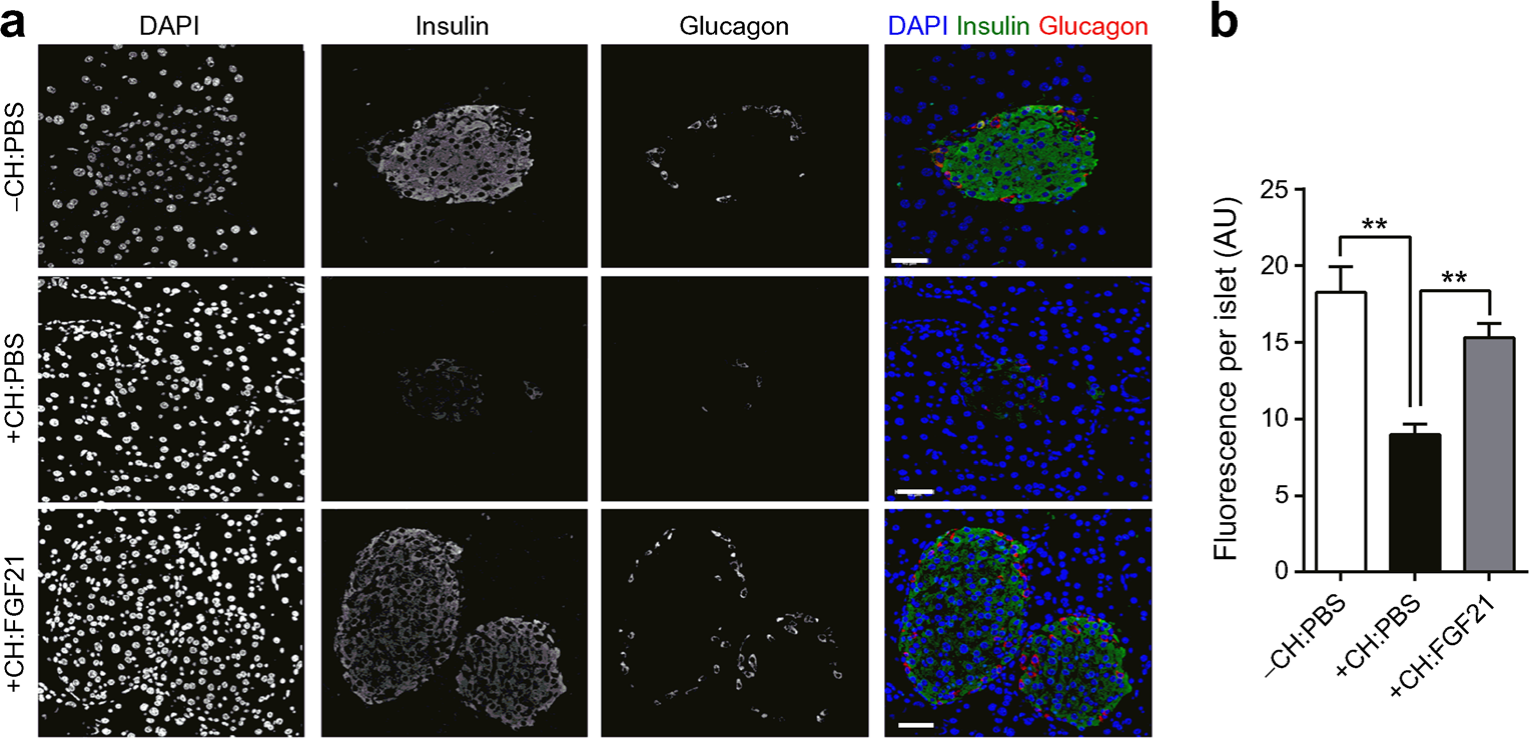
Carbohydrate feeding for 2 weeks attenuates pancreatic islet integrity which is improved by FGF21 treatment. Mice were treated as described in Fig. 2. (**a**) Staining of insulin (green), glucagon (red) and nuclei (blue) in pancreatic slices. Scale bars, 40 μm. (**b**) Quantified insulin content of the staining. Data are presented as mean ± SEM (*n* = 3 [ten islets/animal]/group). Differences compared with +CH:PBS group were calculated by one-way ANOVA (**b**). **p < 0.01

Histological analysis of the pancreatic islets at the end of the study revealed substantial islet destruction in PBS-treated +CH-fed mice, while PBS-treated −CH-fed mice displayed a normal islet morphology as expected and shown earlier [35, 36] (Fig. 4a). FGF21-treated +CH mice developed an intermediate phenotype with no disruption of normal islet cytoarchitecture but numerically smaller amounts of synthesised insulin (Fig. 4a, b).

Final liver weight was significantly higher in +CH-fed compared with −CH-fed mice treated with PBS, probably caused by a trend of increased hepatic triacylglycerol (*p* = 0.10; ESM Fig. 2). While gWAT mass was significantly lower (*p* < 0.05) in FGF21-treated mice compared with PBS-treated mice on the +CH diet, mass of BAT was highest in FGF21-treated +CH-fed mice (*p* < 0.05; ESM Fig. 2a). In summary, without changes in total fat mass, FGF21 prevents hyperglycaemia and improves glucose clearance in NZO mice fed a carbohydrate-containing diet.

### FGF21 increases EE in NZO mice

**Fig. 5:**
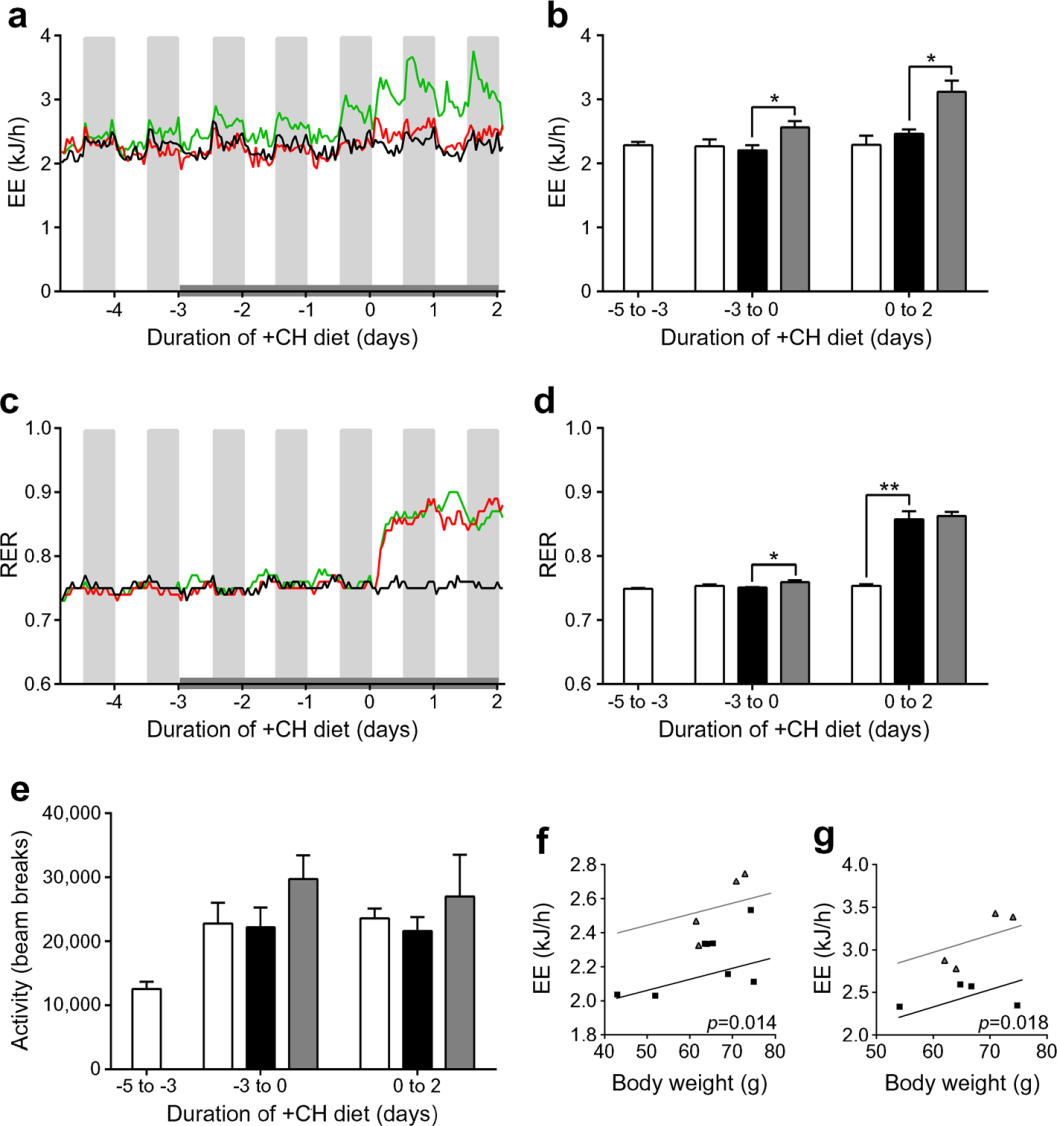
FGF21 increases EE in NZO mice. Mice were treated as described in Fig. 2. Animals were treated s.c. with PBS or rhFGF21 (1 μg/g body weight) starting 3 days before the diet switch and ending 2 days after the diet switch (indicated by the grey bar). (**a**) EE in NZO mice and (**b**) average EE over different periods of time. (**c**) RER, (**d**) average RER and (**e**) activity (beam breaks) over different periods of time. Black line/white bars, −CH:PBS; red line/black bars, +CH:PBS; green line/grey bars, +CH:FGF21. Scatter plots illustrating the relationship between body weight and EE, based on ANCOVA, in NZO mice treated with PBS or rhFGF21 during (**f**) the first 3 days of FGF21 treatment (day −3 until 0) and (**g**) days 1 to 2 (black squares, PBS; grey triangles, FGF21). Data are presented as mean ± SEM (*n* = 4/group). Differences compared with +CH:PBS group were calculated by one-way ANOVA. *p < 0.05, **p < 0.01

Studies show that pharmacological FGF21 treatment acts both in the brain and directly on adipose tissue to increase EE, stimulate sympathetic outflow and upregulate thermogenic markers in BAT and WAT [14, 17, 41, 42]. Besides increased BAT mass following FGF21 treatment (ESM Fig. 2a), FGF21-treated NZO mice showed an increased EE (*p* < 0.05) on the −CH diet and an even higher EE (*p* < 0.05) on the +CH diet compared with PBS-treated animals (Fig. 5a, b). Analysis of the EE data using ANCOVA with body weight as the covariant demonstrated an FGF21-dependent increase in EE (Fig. 5f, g). The RER was significantly increased by the +CH diet in both PBS- and FGF21-treated mice, which reflects the expected increase in the rate of carbohydrate oxidation (*p* < 0.01; Fig. 5c, d). In contrast, there were no effects of either diet or PBS and FGF21 treatment on locomotor activity (Fig. 5e). As shown before (Fig. 3b), FGF21 treatment increased food intake in mice on the −CH diet (*p* < 0.01; ESM Fig. 3a).

### FGF21 induces thermogenic and lipogenic genes in sWAT and BAT in NZO mice

**Fig. 6:**
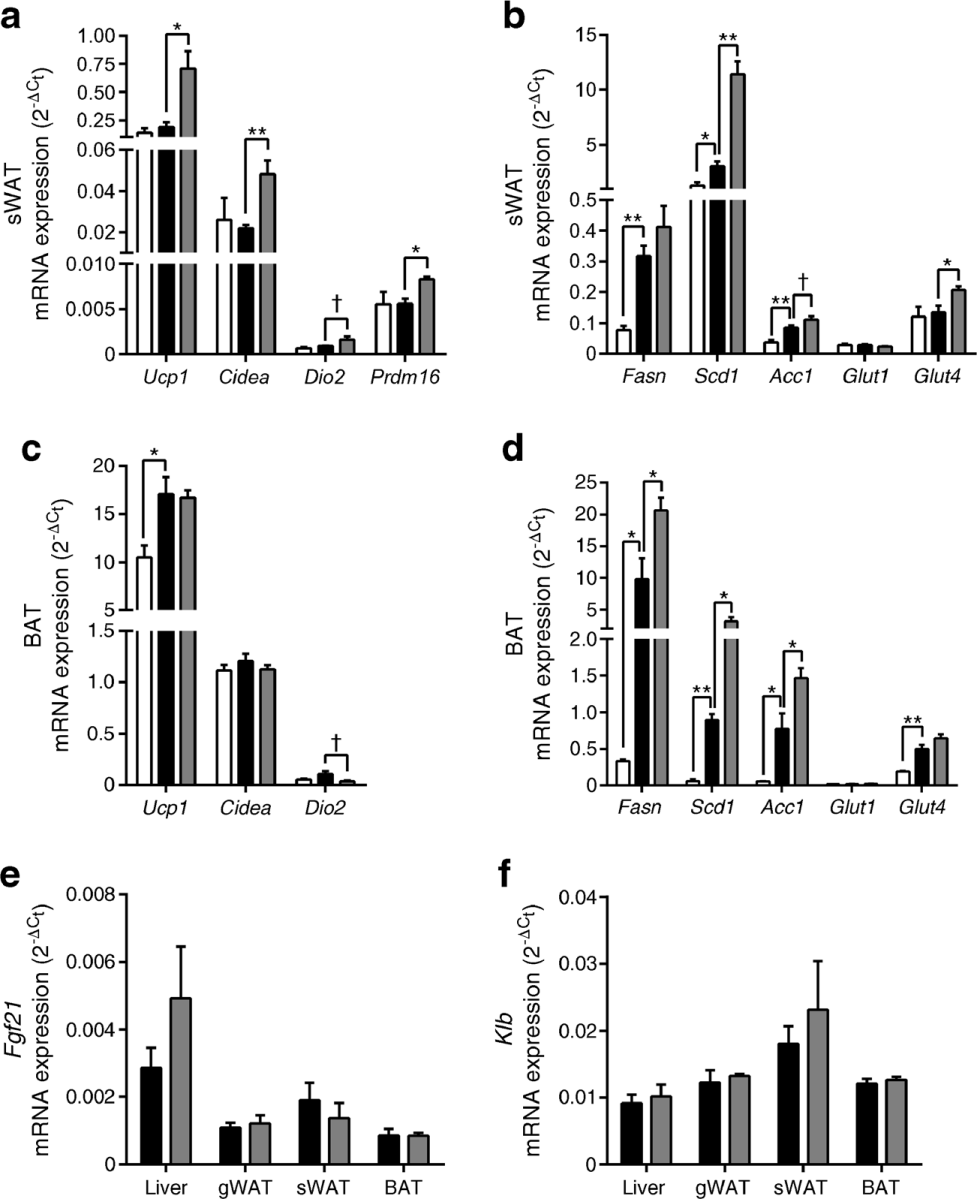
Effect of carbohydrate feeding and FGF21 on sWAT and BAT metabolic genes in NZO mice. Tissues were collected from mice that were treated as described in Fig. 2. Animals were treated s.c. with PBS or rhFGF21 (1 μg/g body weight) starting 3 days before the diet switch and ending 2 days after the diet switch. Two days after the diet switch animals fasted for 4 h were killed. Thermogenic genes from (**a**) sWAT and (**c**) BAT, and lipogenic and glucose transporter genes from (**b**) sWAT and (**d**) BAT were measured via real-time PCR. Gene expression of (**e**) *Fgf21* and (**f**) *Klb* in different tissues. White bars, −CH:PBS; black bars, +CH:PBS; grey bars, +CH:FGF21. Data are presented as mean ± SEM (*n* = 4/group). Differences compared with +CH:PBS group were calculated by one-way ANOVA (**a**–**d**) or two-tailed *t* test (**e**, **f**). ^†^0.1 > *p* > 0.05, *p < 0.05, **p < 0.01

We next tested whether the robust increase in EE due to FGF21 was associated with changes of thermogenic markers in BAT and sWAT, which is prone to browning. Consistent with the acute increase in EE induced by FGF21, sWAT *Ucp1*, *Cidea* and *Prdm16* were significantly increased (*p* < 0.05; Fig. 6a) and *Dio2* mRNA expression tended to be higher (*p* = 0.06), which shows strong evidence for browning in sWAT. There was no effect of the +CH diet itself on sWAT *Ucp1*, *Cidea*, *Dio2* or *Prdm16* mRNA expression (Fig. 6a). Feeding carbohydrate increased the mRNA expression of genes associated with lipogenesis within the sWAT (*Fasn*, *Scd1*, *Acc*; *p* < 0.05; Fig. 6b), whereas FGF21-treated +CH mice showed even significantly higher *Scd1* (*p* < 0.01) and *Acc* mRNA expression by trend (*p* = 0.08) compared with the PBS-treated +CH mice. Interestingly, in sWAT the expression of *Glut1* was not induced by FGF21, instead *Glut4* mRNA expression was highest in FGF21-treated +CH mice (*p* < 0.05; Fig. 6b).

Interestingly, FGF21 did not induce genes associated with thermogenesis (*Cidea*, *Dio2*) within BAT, but +CH feeding significantly increased *Ucp1* mRNA expression (*p* < 0.05; Fig. 6c). Lipogenic genes (*Fasn*, *Scd1*, *Acc1*) were significantly elevated in response to the +CH diet and significantly highest due to FGF21 treatment in BAT (*p* < 0.05; Fig. 6d). Neither the +CH diet nor FGF21 altered BAT *Glut1* expression but *Glut4* mRNA expression was significantly increased by the +CH diet (*p* < 0.01; Fig. 6d). Finally, exogenous administration of FGF21 did not affect the gene expression of *Fgf21* and *Klb* in the liver, gWAT, sWAT or BAT (Fig. 6e, f).

These data demonstrate that the effects of FGF21 on thermogenesis are intact and presumably engage UCP1-dependent increases in EE.

## Discussion

In this study we have clearly demonstrated for the first time in obese diabetes-susceptible NZO mice with the characteristic trait of beta cell loss that exogenous treatment with FGF21 fully prevents the onset of hyperglycaemia and beta cell loss despite increased food intake without differences in the total fat mass. Recent studies have shown beneficial roles of FGF21 in regulating insulin sensitivity and glucose homeostasis. However, it has also been suggested that obesity is an FGF21-resistant state in the context of effects of endogenous levels of FGF21 [24, 25], indicating the lack of a benefit by FGF21 exposure in obese models. It is well-known that FGF21, which is primarily secreted from the liver and functions in multiple tissues, is a key endocrine regulator of glucose, lipid and energy metabolism. FGF21 activates FGFRs in the context of co-receptor β-klotho expression. After that, FGF21 alters ERK1/2 phosphorylation and stimulates glucose uptake. Pharmacologically administered FGF21 mediates its effects via FGF21 receptors, but individual endpoints of FGF21 pharmacology appear to be mediated by distinct downstream factors, such as adiponectin or leptin [23]. As shown by Lin et al [43], metabolic effects of FGF21 on glucose homeostasis and insulin sensitivity in mice are mediated by adiponectin.

As expected, NZO mice show increased FGF21 levels in particular when blood glucose levels rise. Consistent with the previously reported ability of FGF21 administration to improve hyperglycaemia and enhance insulin sensitivity and glucose clearance [19, 20, 26, 44], we observed a protection from type 2 diabetes and beta cell loss independent of body fat. The effects are mediated (1) by improving insulin sensitivity and glucose clearance and (2) increasing the EE via browning of sWAT, which might increase glucose use via elevated *Glut4* expression.

Despite a robust loss of lean mass, FGF21 treatment resulted in an improved glucose clearance supported by the Matsuda Index, which indicates improved insulin sensitivity mainly of the skeletal muscle and adipose tissue. This is supported by data showing that *Glut4* mRNA expression is elevated in adipose tissue. Improved insulin sensitivity has already been demonstrated by hyperinsulinaemic–euglycaemic clamp studies in B6-mice treated with FGF21, showing improvement in whole body insulin sensitivity, which is accounted for an increase in glucose uptake into skeletal muscle and adipose tissue [45]. As a consequence, FGF21-treated carbohydrate-fed mice show normal blood glucose levels.

In addition to the prevention of hyperglycaemia, which itself suppresses the beta cell loss, pancreatic islets are protected from glucolipotoxicity and cytokine-induced apoptosis by FGF21 treatment [19]. Since no effect of FGF21 on islet cell proliferation has been observed, it was concluded that preservation of beta cells and their functioning by FGF21 may contribute to the beneficial effects of FGF21 on glucose homeostasis [19]. We can speculate that the protective effect of FGF21 is not influencing the islet cytoarchitecture directly and that it is a secondary response to a prevention of glucolipotoxicity.

As recently shown in NZO mice [36], treatment with a glucagon-like peptide 1–oestrogen hybrid decreased food intake resulting in body weight loss (mainly body fat), preserved normoglycaemia, improved glucose tolerance and beta cell protection. FGF21 treatment improved glucose homeostasis without reducing daily food intake; rather, animals treated with FGF21 consumed more food when caloric intake was normalised to animal body weight. This is in concordance with earlier observations made in FGF21 transgenic mice [20] and after FGF21 administration [17, 26, 46], while no effect on food intake was observed in other studies [14, 41]. In particular, Coskun and colleagues provided evidence that the effects of FGF21 on blood glucose levels and body weight are decoupled [26]. It is unclear if FGF21 is modulating food intake directly or if the hyperphagic response is secondary to the increased EE and a compensatory response to resist weight loss.

It was demonstrated in recent studies that pharmacological FGF21 treatment acts both in the brain and directly on adipose tissue to increase EE, stimulate sympathetic outflow and upregulate thermogenic markers in BAT and WAT [14, 17, 26, 41, 42]. Consistent with the previous findings in other animal models [14, 17, 26, 41, 46], FGF21 treatment in NZO mice increased the overall EE with comparable magnitudes during both dark and light cycles. Interestingly, EE was further elevated by FGF21 after switching to the carbohydrate-containing diet. Eventually, the increase in EE due to FGF21 is not caused by an increase in physical activity in these animals, which is in contrast to other studies demonstrating increased metabolic rate along with increased physical activity [46]. FGF21 is sufficient to stimulate browning of sWAT as assessed by mRNA expression of *Ucp1* and the induction of gene expression associated with browning (*Cidea*, *Dio2*, *Prdm16*). This outcome is consistent with evidence that pharmacological FGF21 treatment increases EE, upregulates UCP1 and promotes sWAT browning [17, 41, 42, 47, 48]; without changing the gene expression of the FGF21 co-receptor β-klotho (*Klb*). This suggests that in diabetic mice the downregulation of *Klb* in BAT may not be FGF21 dependent.

Taken together, the above experiments produce four notable conclusions. (1) Consistent with recent studies showing that FGF21 improves glucose homeostasis in various obese mice models, we demonstrate superior efficacy of FGF21 in preventing the onset of diet-induced diabetes in male NZO mice. (2) This protective effect is not caused by hypophagia (NZO mice are hyperphagic) or loss of fat mass, but (3) rather by an increase in EE due to the browning of sWAT and more directed storage of lipids in fat tissue. Finally, (4) these data indicate that obesity does not induce FGF21 resistance in NZO mice. As such, these findings support the diabetes-susceptible NZO mouse as a potential animal model to study endogenous FGF21 actions with regard to the prevention of diabetes.

## Electronic supplementary material

ESM (PDF 224 kb)

## Abbreviations

BAT: Brown adipose tissue
−CH: Carbohydrate-free high-fat
+CH: Carbohydrate-containing high-fat
EE: Energy expenditure
FGF: Fibroblast growth factor
FGFR: Fibroblast growth factor receptor
gWAT: Gonadal white adipose tissue
NZO: New Zealand obese
RER: Respiratory exchange ratio
sWAT: Subcutaneous white adipose tissue
WAT: White adipose tissue
