# Supplementary material for: FGF21 improves glucose homeostasis in an obese diabetes-prone mouse model independent of body fat changes
Source: Diabetologia. 2017 Aug 2;60(11):2274–84. doi: 10.1007/s00125-017-4389-x (PMC6448882; doi:10.1007/s00125-017-4389-x)
Supplement: Supplementary file 1 — (PDF 224 kb) [file 125_2017_4389_MOESM1_ESM.pdf]

ESM Table 1: Composition of diets

Ingredient	- CH (#105789, Altromin; Lage, Germany)
	%, w/w
Crude protein	32.1
Crude fat	30.6
Crude fibre	20.2
Crude ash	5.6
Moisture	4.2
N-free extract	7.3

Ingredient	+ CH (self-made)	
	%, w/w	Manufacturer
Casein	20	Bayerische Milchindustrie eG; Landshut, Germany
Palm fat	13.5	Ostthüringer Nahrungsmittelwerk; Gera, Germany
Margarine	13.5	Ostthüringer Nahrungsmittelwerk; Gera, Germany
Safflower oil	0.5	Kunella Feinkost; Cottbus, Germany
Linseed oil	0.5	Kunella Feinkost; Cottbus, Germany
Microcellulose	5	J. Rettenmaier und Söhne; Ellwangen-Holzmühle, Germany
Mineral mixture	5	Altromin; Lage, Germany
Vitamin mixture	2	Altromin; Lage, Germany
Sucrose	10	Pfeifer & Langen KG; Cologne, Germany
Starch	30	Kröner Stärke; Ibbenbüren, Germany

Macronutrient content	- CH	+ CH
Energy density (kJ/g)	16.8	21.9
Protein (% of total energy)	31	16.2
Carbohydrates (% of total energy)	0	32.4
Fat (% of total energy)	68	51.4

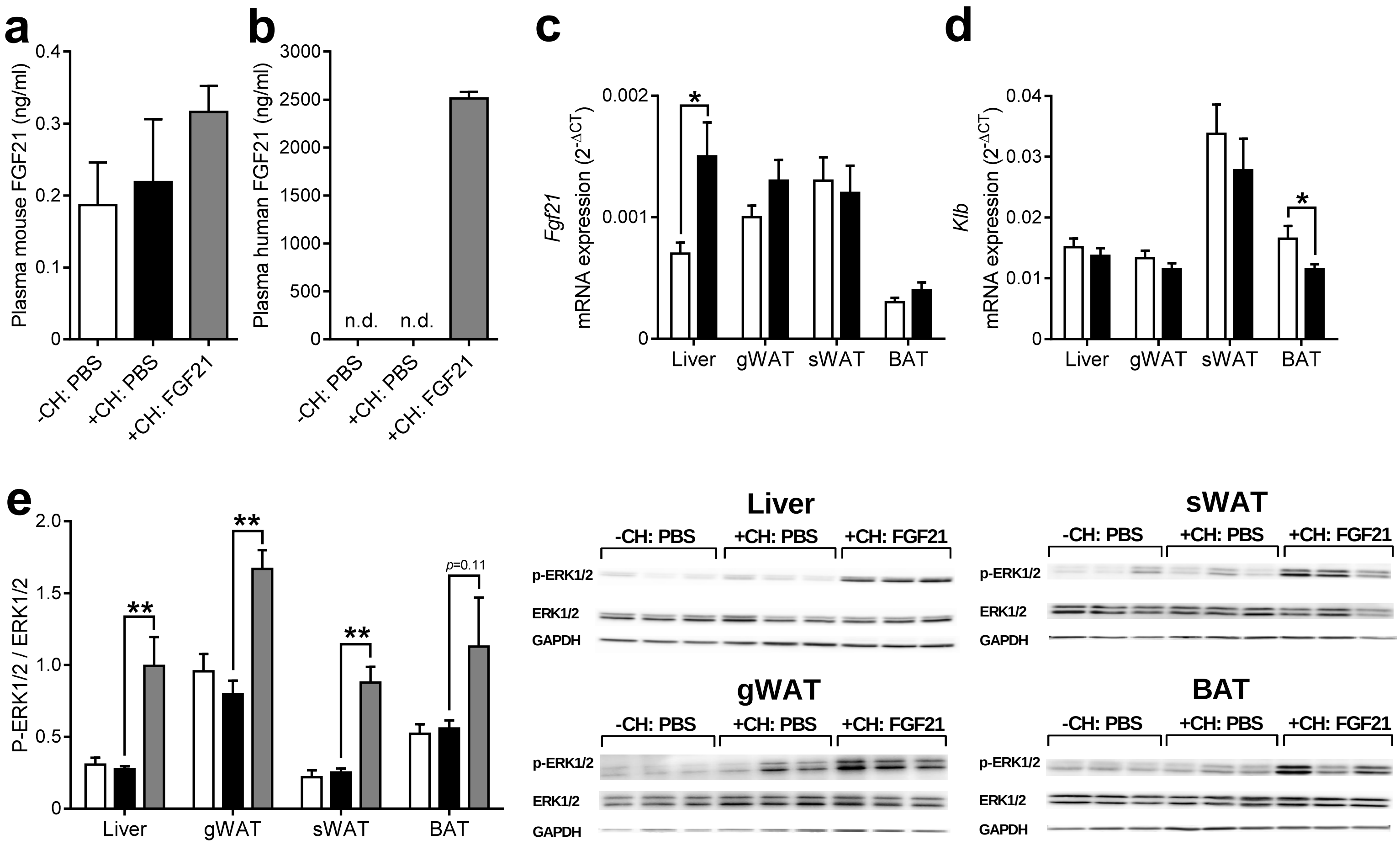

ESM Figure 1. FGF21 stimulates phosphorylation of ERK1/2 in liver and fat of NZO mice. At the age of 5 weeks, NZO mice were placed on a carbohydrate-free high-fat diet (-CH) for 13 weeks, at which point a random subgroup of animals was transferred to a carbohydrate-containing high-fat diet (+CH) for 2 weeks. (**a**) Random plasma FGF21 concentrations 3 days before the diet switch and (**b**) plasma human FGF21 one hour after sc injection at day 0. Gene expression of (**c**) *Fgf21* and (**d**) *Klb* in liver, gWAT, sWAT, and BAT of NZO mice 14 days after the diet switch.14 days after the diet switch, 4-hour fasted animals were treated subcutaneously with PBS or rhFGF21 (1 µg/BW g) 30 min before sacrifice. (**e**) Western blot of ERK1/2 phosphorylation in liver, gWAT, sWAT, and BAT. White bars, -CH: PBS; black bars, +CH: PBS; grey bars, +CH: FGF21. Data are presented as mean ± SEM (n = 6-7/group). Differences compared with +CH:PBS group were calculated by one-way ANOVA (**a**, **b**, **e**) and two-tailed t-test (**c**, **d**), respectively. *$p < 0.05$; **$p < 0.01$.

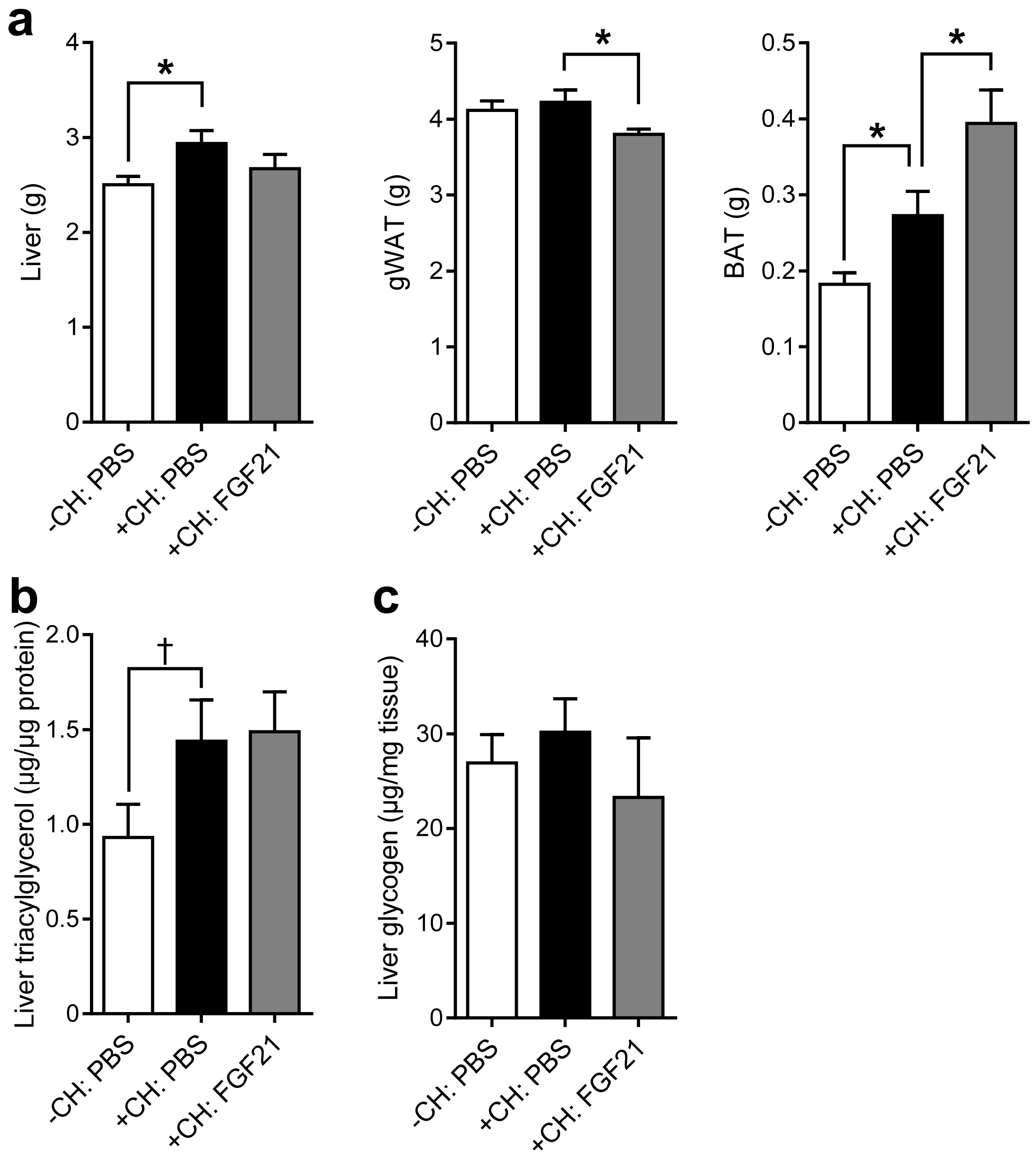

ESM Figure 2. FGF21 modifies changes in organ mass induced by carbohydrate feeding in NZO mice. At the age of 5 weeks, NZO mice were placed on a carbohydrate-free high-fat diet (-CH) for 13 weeks, at which point a random subgroup of animals was transferred to a carbohydrate-containing high-fat diet (+CH) for 2 weeks. Animals were treated subcutaneously with PBS or rhFGF21 (1 µg/BW g) starting 3 days before the diet switch and ending 7 days after the diet switch. 14 days after the diet switch, 4-hour fasted animals were sacrificed. Weight of (**a**) liver, gWAT, and BAT. (**b**) Liver triacylglycerol and (**c**) glycogen content. Data are presented as mean ± SEM (n = 6-7/group). Differences compared with +CH:PBS group were calculated by one-way ANOVA. $^{\dagger}0.1 > p > 0.05$; $^{*}p < 0.05$.

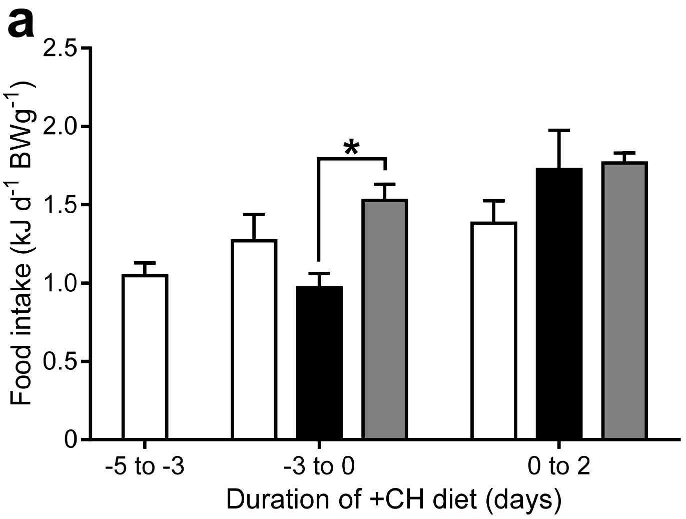

ESM Figure 3. FGF21 transiently increases food intake in NZO mice. At the age of 5 weeks, NZO mice were placed on a carbohydrate-free high-fat diet (-CH) for 13 weeks, at which point a random subgroup of animals was transferred to a carbohydrate-containing high-fat diet (+CH) for 2 days. Animals were treated subcutaneously with PBS or rhFGF21 (1 µg/BW g) starting 3 days before the diet switch and ending 2 days after the diet switch. (**a**) Food intake over different periods of time. White bars, -CH: PBS; black bars, +CH: PBS; grey bars, +CH: FGF21. Data are presented as mean ± SEM (n = 4/group). Differences compared with +CH:PBS group were calculated by one-way ANOVA. ** $p < 0.01$.
